# High-Efficiency Removal of Lead and Nickel Using Four Inert Dry Biomasses: Insights into the Adsorption Mechanisms

**DOI:** 10.3390/ma16134884

**Published:** 2023-07-07

**Authors:** Candelaria Tejada-Tovar, Angel Villabona-Ortíz, Ángel Darío González-Delgado

**Affiliations:** 1 Process Design and Biomass Utilization Research Group (IDAB), Chemical Engineering Department, Universidad de Cartagena, Avenida del Consulado St. 30, Cartagena de Indias 130015, Colombia; ctejadat@unicartagena.edu.co (C.T.-T.); avillabonao@unicartagena.edu.co (A.V.-O.); 2 Nanomaterials and Computer Aided Process Engineering Research Group (NIPAC), Chemical Engineering Department, Universidad de Cartagena, Avenida del Consulado St. 30, Cartagena de Indias 130015, Colombia

**Keywords:** adsorption mechanism, bioadsorption, heavy metal ions, kinetics, isotherms, wastewater treatment

## Abstract

In this study, inert dry bioadsorbents prepared from corn cob residues (CCR), cocoa husk (CH), plantain peels (PP), and cassava peels (CP) were used as adsorbents of heavy metal ions (Pb^2+^ and Ni^2+^) in single-batch adsorption experiments from synthetic aqueous solutions. The physicochemical properties of the bioadsorbents and the adsorption mechanisms were evaluated using different experimental techniques. The results showed that electrostatic attraction, cation exchange, and surface complexation were the main mechanisms involved in the adsorption of metals onto the evaluated bioadsorbents. The percentage removal of Pb^2+^ and Ni^2+^ increased with higher adsorbent dosage, with Pb^2+^ exhibiting greater biosorption capacity than Ni^2+^. The bioadsorbents showed promising potential for adsorbing Pb^2+^ with monolayer adsorption capacities of 699.267, 568.794, 101.535, and 116.820 mg/g when using PP, CCR, CH, and CP, respectively. For Ni^2+^, Langmuir’s parameter had values of 10.402, 26.984, 18.883, and 21.615, respectively, for PP, CCR, CH, and CP. Kinetics data fitted by the pseudo-second-order model revealed that the adsorption rate follows this order: CH > CP > CCR > PP for Pb^2+^, and CH > CCR > PP > CP for Ni^2+^. The adsorption mechanism was found to be controlled by ion exchange and precipitation. These findings suggest that the dry raw biomasses of corn cob residues, cocoa husk, cassava, and plantain peels can effectively remove lead and nickel, but further research is needed to explore their application in industrial-scale and continuous systems.

## 1. Introduction

Agroindustrial activities generate a significant number of residues, which can pose a challenge for sustainable waste management. These residues arise from different processes, such as crop cultivation, harvesting, processing, and packaging [1]. They can be composed of organic matter, plant debris, and agrochemicals. The proper disposal of these residues is crucial to minimizing their negative impact on the environment and human health. Improper handling can lead to soil and water contamination, air pollution, and greenhouse gas emissions. However, these residues can also be a valuable source of renewable energy and bio-based products through their conversion into biogas, biofuels, and other value-added products [2]. Therefore, the development of efficient and sustainable waste management strategies for agroindustrial residues is critical to promoting environmental sustainability and circular economy practices.

Reusing residues from agroindustrial activities as bioadsorbents is gaining attention due to its potential to reduce waste and promote the circular economy. Many agroindustrial processes generate significant amounts of residues, which are often considered a problem for disposal. However, these residues can also be a valuable resource if properly utilized. By converting them into bioadsorbents, they can be used to remove contaminants from wastewater, air, and soil while also reducing the need for synthetic adsorbents. This approach promotes the principles of zero waste and the circular economy, which aim to minimize waste generation and maximize resource utilization [3].

Heavy metal contamination refers to the presence of toxic elements such as lead, mercury, cadmium, and arsenic in the environment, usually as a result of human activities such as industrial processes, mining, and improper disposal of waste [4]. This contamination can seriously affect human health and the ecosystem, including neurological damage, cancer, and reduced plant growth [5]. Some of the most dangerous heavy metals present in water include lead, mercury, cadmium, arsenic, and chromium. Exposure to these metals can cause serious health problems, such as cancer, neurological disorders, organ damage, and developmental abnormalities [6]. They can also accumulate in the food chain, posing a risk to animals and humans who consume contaminated food or water [7]. Therefore, it is important to monitor and prevent the contamination of water sources with these hazardous materials.

Lead can enter water supplies through corroded pipes and plumbing systems, as well as through industrial waste and mining runoff. The effects of lead exposure can be devastating for humans, animals, and the environment [8]. It can cause serious health problems such as brain damage, reduced cognitive function, anemia, and impaired growth and development [9]. For animals, lead exposure can lead to reduced fertility and weakened immune systems [10]. Lead pollution can also have long-lasting effects on the environment, including damage to soil quality and water sources. Therefore, it is important to implement measures to prevent lead pollution and mitigate its harmful effects.

Nickel is a toxic heavy metal that can contaminate water sources through industrial activities, the natural weathering of rocks, and soil erosion [11]. Exposure to high levels of nickel can cause adverse health effects in humans, such as skin irritation, respiratory problems, kidney damage, and even cancer [12,13]. Nickel also affects animals, disrupting their growth and reproduction. Additionally, the accumulation of nickel in the environment can lead to ecosystem disturbances and affect plant growth and biodiversity [14]. Therefore, monitoring and regulating nickel concentrations in water sources is crucial to mitigating negative impacts on human health and the environment.

Several physicochemical methods are employed to remove heavy metals from aqueous solutions, including solvent extraction [15], ion exchange [16], chemical precipitation [17], photoelectrocatalytic methods [18], electrocatalytic methods [19], reverse osmosis [20], and membrane separation [21]. However, certain techniques necessitate energy consumption, chemical additives, and face limitations when dealing with concentrations below 60 mg/L [18,22]. Consequently, there is a significant need to develop efficient and cost-effective separation processes.

Accordingly, bioadsorption offers a viable alternative to achieve the desired objective, which necessitates the development of new adsorbents with efficient adsorption capacities [23]. The utilization of biomass derived from agricultural residues as adsorbents has been extensively investigated due to its low cost, immediate availability, and high removal efficiency. Various bioadsorbents, including wood-derived biochar [24], plantain peels [22,25], Nitrile amidoximation of manna tree [26], Date palm biochar [27], rice husk [28], Carbon from cassava peels activated with KOH [29], Peat moss [30], potato husks [31], lemon [32], coffee pulp [33], and other residues, have been tested for the removal of these types of contaminants [34,35].

In this study, the efficiency of the raw biomasses of plantain peels (PP), corn cob residues (CCR), cocoa husk (CH), and cassava peels (CP) was evaluated for removing three heavy metals (lead and nickel) from aqueous solutions. These residues were considered precursors of bioadsorbents. Colombia is an agricultural country where a wide variety of crops are harvested. Banana production in Colombia is estimated at 1.7 million tons per year, of which approximately 40% by mass is peel, resulting in the generation of around 680,000 tons of banana peel per year [36]. Additionally, in the country, around 1.9 million tons of traditional yellow corn are produced annually, of which 30% by mass is a residue known as corn cobs or corn husks, amounting to 570,000 tons per year [37]. Regarding cocoa, in Colombia, 62,158 tons were produced in 2022, of which the husk accounts for between 52% and 70% of the wet weight of the fruit, amounting to approximately 32,300–43,500 tons of this residue [38]. In the country, around 400,000 tons of cassava peels are generated annually as a residue from the 2 million tons collected, which represents 20% of its weight [39].

Thus, the effects of adsorption time, pH, dosage, particle size, temperature, and initial ion concentration were studied in detail. Other parameters such as distribution coefficient, adsorption isotherm, kinetics, and statistical analysis of the adsorption process were also evaluated. The adsorption capacities of these biomasses were assessed by Langmuir, Freundlich, Dubinin- Radushkevich (D-R), Redlich-Peterson (R-P), and Temkin adsorption isotherm models. The main objectives of the present study were: (1) to evaluate the adsorption capacity of the bioadsorbents under study for the removal of two specific heavy metals (lead and nickel); and (2) to identify the adsorption mechanisms involved in the adsorption of the heavy metals.

## 2. Materials and Methods

### 2.1. Materials

The reagents used in this study were of analytical grade (purity 99.999% on a trace metal basis) and provided by Sigma Aldrich (St. Louis, MO, USA). The biomaterials under study were obtained as byproducts of agroindustrial activities in the department of Bolivar (Colombia). Pictures of the raw biomasses are shown in Supplementary Materials (Figure S1).

### 2.2. Biomass Pretreatment

Initially, the biomasses were washed and dried at 60 °C until they reached constant mass. Then, they were ground to particles of 0.355 mm, 0.5 mm, and 1 mm.

### 2.3. Physicochemical Characterization

The acid or basic groups were determined by Boehm titration. Infrared analyses were performed using an IRAinfinity-1Fourier transform spectrophotometer. The samples were placed in the equipment cell as a powder without previous treatment, making 32 scans in the range of 400–4000 cm^−1^, using 100 mg KBr pellets as a reference. The physical characteristics of the adsorbents and their elemental composition identification were carried out by SEM-EDX analysis using JEOL JSM-6490 LV equipment. These studies were carried out before and after the metal adsorption assays.

The pH point of zero charge (pH_PZC_) was determined to establish the equilibrium charge of the adsorbent surface. Therefore, water samples with a pH between 3 and 11 were put in contact with 1 g of each bioadsorbent and stirred for 24 h at room temperature. The final pH of each sample was taken to be analyzed by the graphic method to identify the pH_PZC_ of the material [40].

### 2.4. Preparation of Synthetic Solutions

The synthetic water solutions were prepared using a known quantity of Nickel Sulphate (NiSO_4_) and Lead nitrate (Pb(NO_3_)_2_) salts at a concentration of 100 ppm, and the different pH values established in the experimental design were adjusted using 2 M Sodium Hydroxide (NaOH) and 2 M Hydrochloric Acid (HCl) solutions.

### 2.5. Batch Experiments for the Removal of Ni^2+^ and Pb^2+^

Adsorption experiments were carried out in batch mode. The effect of the pH was studied by placing into an Erlenmeyer flask with 0.5 g in contact with 100 mL of Pb^2+^ and Ni^2+^ solutions at 100 mg/L and at different values of pH (2, 4, and 6) and stirring constantly at room temperature (298 K), 200 rpm, for 24 h with a volume of 100 mL. Then, at the best pH condition, the effect of temperature, particle size, and adsorbent dose on the adsorption performance was investigated following the design of experiments presented in Table 1. The central composition design implemented in this study consisted of 16 experiments and assessed the errors related to the experimental procedure and the analysis of the data by conducting duplicate tests at the central point for the variables under study. The statistical analysis was conducted using Statgraphics Centurion XIX software (Plains, VA, USA). The size classification was carried out using mechanical agitation in a sieve shaker of the Edibon Orto Alresa brand, using sieves with mesh numbers 120, 45, 35, 18, and 16, according to the ASTM [41], in order to select the sizes to evaluate.

After the experiments, the final concentration of remaining metal in solution was determined by atomic absorption spectrophotometry using an ICE 3000 instrument at 217 nm and 232 nm for Pb^2+^ and Ni^2+^, respectively. The batch system adsorption efficiency and adsorption capacity were calculated from the initial and final concentrations of the samples using Equations (1) and (2), respectively.

(1) $$M[\%]=\frac{C_{i}-C_{f}}{C_{i}}\times 100$$

(2) $$q_{t}=\frac{C_{i}-C_{f}}{m}\times V$$

where *q_t_* is the adsorption capacity of the adsorbent (mg/g); *C_i_* and *C_f_* (mg/L) are the metal ion concentrations in the solution initially and at the end of the experiments, respectively; *V* is the solution volume (L); and m is the mass of the adsorbent (g).

### 2.6. Study of the Effect of Time and the Initial Concentration

At the best conditions obtained, i.e., those values of temperature, particle size, and adsorbent dose that presented the highest removal percentage for each metal and biomass, kinetic studies were carried out by taking samples at different time intervals over a total of 24 h. The results obtained in each of the kinetic tests were adjusted to the kinetic models of pseudo-first order, pseudo-second order, Elovich, and intraparticle diffusion by non-linear fitting in the OriginPro 2023 software (Northampton, MA, USA) to understand the behavior of Pb^2+^ and Ni^2+^ adsorption with each adsorbent over time and determine the mechanism by which the adsorption process occurs in each of these systems. The study of adsorption isotherms was carried out by varying the initial concentration at the best experimental conditions obtained from the execution of the experimental design in Table 1 and adjusting the experimental data to the Langmuir, Freundlich, Dubinin-Radushkevich (D-R), Redlich-Peterson (R-P), and Temkin models.

## 3. Results

### 3.1. Characterization of the Biomasses

The bioadsorption technology implies a complex phenomenon with several steps; therefore, many factors affect this phenomenon. Among these, the surface chemistry of the studied biomasses has a considerable impact on the process. Thus, the surface of the bioadsorbents was studied by Böehm titration and pH point of zero charge, as well as FTIR and SEM-EDX, before and after the removal of Pb (II) and Ni (II).

#### Surface Properties

The pH point of zero charge (pH_PZC_) defines the pH at which the net surface charge is zero; at this pH, the adsorbent’s surface neither attracts nor repels ions, and it exhibits minimal reactivity. The pH_PZC_ is an essential parameter for characterizing adsorbents’ surface properties, particularly for the design and optimization of adsorption processes [42].

The pH_PZC_ is found to be 4.29, 4.79, 4.65, and 5.92 for PP, CCR, CP, and CH, respectively. The above results are coherent with the quantification of the surface functional groups obtained via the Boehm method shown in Table 2, considering that pH_PZC_ depends on the type of functional groups present in the amphoteric surface of bioadsorbents [43]. Therefore, the surface is positively charged for pH values below pH_pzc_ and negatively charged for higher pH values. For red beet peel, a pH_PZC_ of 5.48 was obtained, indicating that at pH < 5.48, the surface of the biomass will be positively charged and the affinity with the anions will be higher [43]. Further, similar results have been reported for banana peels (pH_PZC_ 5.63), gibto seed peel (4.3) [44], pumpkin peel biochar (5.5) [45], and groundnut husk modified with Guar Gum (7.0) [46].

Note: The analysis was carried out in triplicate; the standard deviation of results is ±0.002.

**Figure 1:**
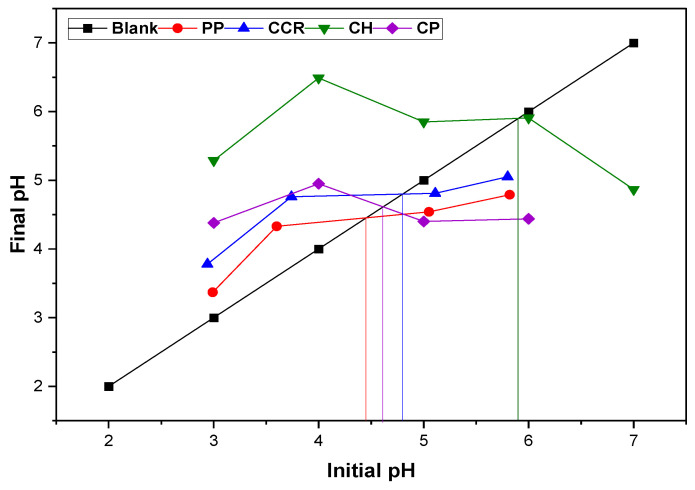
pH_PZC_ for the bioadsorbents under study; vertical lines are the intercept points with the blank.

Böehm titration is used to determine the surface acidity of a bioadsorbent. The method involves titrating the sample with different basic solutions and measuring the amount of base required to neutralize the surface acidic groups; the results are expressed as the total acidity of the surface in milliequivalents per gram. This method allows one to understand the surface chemistry and reactivity of the material, which are important for bioadsorption processes [47]. According to the results in Table 2, it is observed that the bioadsorbents have more acidic groups due to the presence of carboxylic and phenolic species in their structure and no lactone groups, which is attributed to the lignocellulosic nature of the biomasses. These results are coherent with the pH_PZC_ values presented in Figure 1, which are proportional to the acidic and basic functions. Similar results were presented for cassava tubes, with zero lactone groups [29]. For a biochar from oil palm fiber, it was reported values of 1.65, 0.2, and 0.15 mmol/g of carboxylic, phenolic, and lactone groups, respectively; this appearance of lactone groups decreases the quantity of the basic functions due to the carbonization phenomenon [48]. For pomegranate peel, it has been reported that the concentration of the acidic functions is more important on its surface [49].

**Figure 2:**
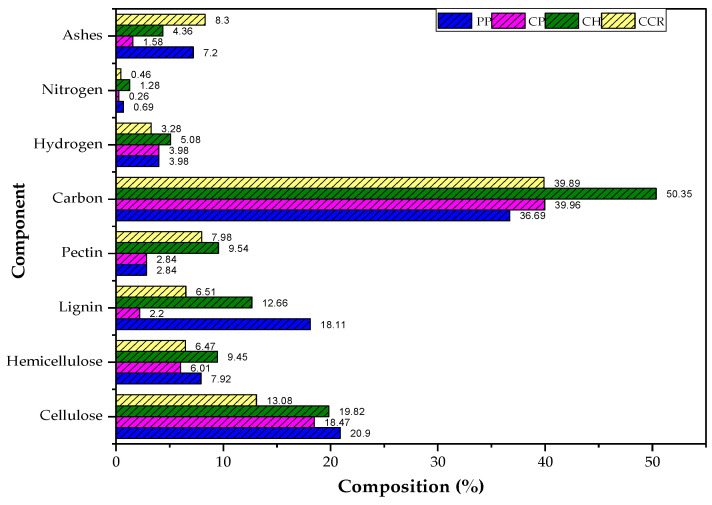
Bromatological analysis for selected biomasses.

Figure 2 displays the results of the bromatological analysis conducted on the bioadsorbents. Carbon was found to be the primary component, followed by hydrogen, which aligns with the expected composition considering the presence of these elements in cellulose, hemicellulose, and lignin molecules. Notably, PP and CH peels exhibited the highest cellulose and hemicellulose content, suggesting potential enhanced removal capabilities during adsorption experiments. The successful removal of heavy metal ions using these lignocellulosic materials can be attributed to the functional groups present, such as hydroxyl, carboxyl, and amines. Therefore, the identification of these functional groups in bioadsorbents holds significant importance [50].

**Figure 3:**
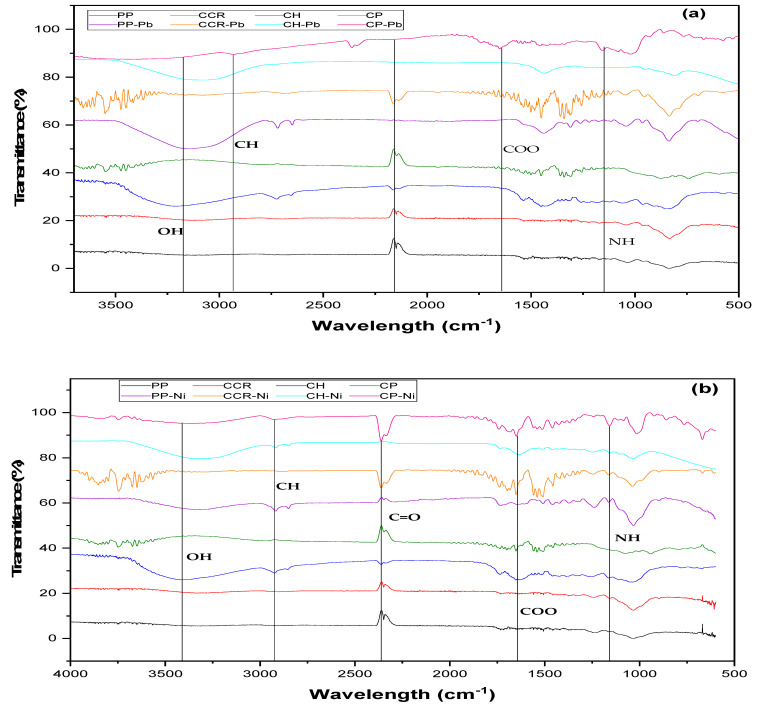
FTIR spectra of bioadsorbents before and after the adsorption of (**a**) Pb^2+^ and (**b**) Ni^2+^.

Fourier Transform Infrared (FTIR) analysis is a technique used to study the functional groups present in bioadsorbents through the peaks at characteristic frequencies that correspond to different functional groups [51]. The FTIR of the bioadsorbents indicated the presence of various functional groups, as shown in Figure 3. The peaks around 3500–3000 cm^−1^ indicate the presence of hydroxyl groups associated with the lignocellulosic nature of the biomasses [52]. There is an observed stretching of the alkyl group (C-H) nearly to 2900 cm^−1^ [53]. C=O stretching of aldehydes or carboxylic acids were evidenced in the peaks around 2170 cm^−1^ [54]. The –COO symmetric stretching is exhibited by peaks around 1600 cm^−1^, and the C–O stretching of esters or ethers and N–H deformation of amine are related to bands near 1000–1350 cm^−1^ [54].

By analyzing the spectra after the adsorption of target molecules, it is possible to identify the functional groups involved in the bioadsorption process and understand the chemical interactions between the bioadsorbent and the adsorbate. It is evidenced by the change in intensity of groups such as hydroxyl, carboxylic, and alkyl. After Ni^2+^ adsorption, there is a peak at 650 cm^−1^ and 774 cm^−1^, which is due to the stretching vibrational peak of the ion [55]. A sharp band of 687 cm^−1^ represents the asymmetric bending vibration of the Pb-O-Pb bond [56].

**Figure 4:**
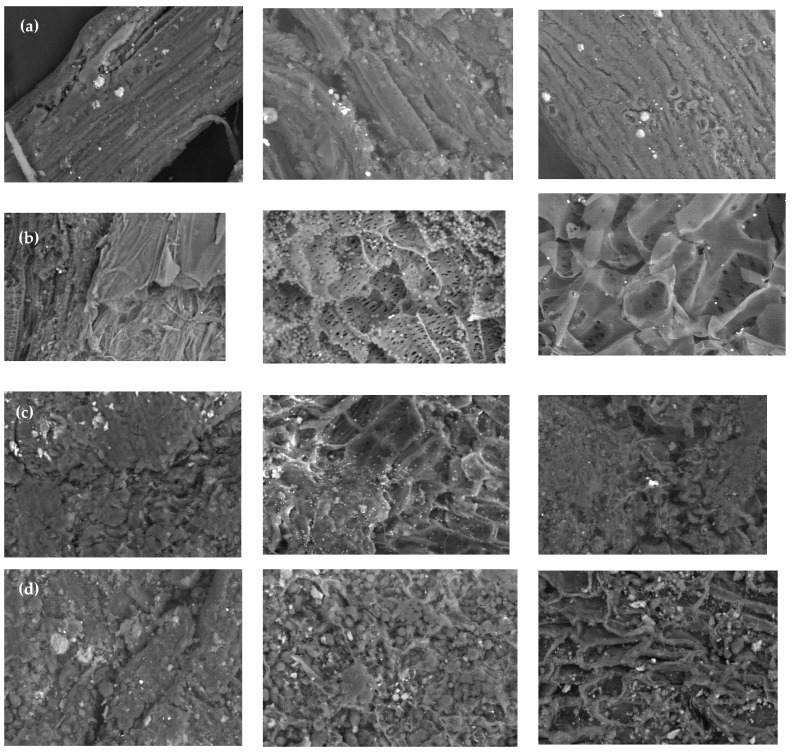
SEM spectra of (**a**) PP, (**b**) CCR, (**c**) CH, and (**d**) CP before (**left**) and after the adsorption of Pb^2+^ (**center**) and Ni^2+^ (**right**) using particle size 0.5 mm and magnification of 500×.

Scanning Electron Microscopy (SEM) coupled with Energy Dispersive X-ray Spectroscopy (EDX) is a powerful technique to investigate the surface morphology, elemental composition, and distribution of bioadsorbents. SEM provides high-resolution images of the surface, while EDX allows for the identification and quantification of the elements present. This analysis can help in understanding the interaction mechanism between the bioadsorbent and the adsorbate as well as identifying any changes that occur in the surface composition before and after the adsorption process [57]. From Figure 4, it is clear that the bioadsorbent surfaces under study were rough, irregular, porous, and compact. The SEM of Pb^2+^ and Ni^2+^ ions loaded biomasses shows a uniform, smooth, and covered surface, which indicates the presence of the ions in the structure of the bioadsorbents. Similar results were found by Afolabi, Musonge, and Bakari [53] when using banana peels to remove Cu^2+^ and Pb^2+^. Altunkaynak, Canoplat, and Yavuz [58] reported that before the adsorption, the orange peel showed well-defined units with irregular morphology, which changed after the Co^2+^ adsorption, presenting agglomerates dispersed over larger crystals. The above matches the changes in the structure of the biomasses under study, which show small changes attributable to the formation of new chemical species between the active centers and the metal ions. The new texture and the crystals observed in the SEM spectra could be attributed to the formation of chelants and precipitations during the removal of the metals from the aqueous solutions [59].

The elemental composition of the PP, CCR, CH, and CP before and after the adsorption was determined by Energy Dispersive X-ray, as shown in Table 3. The presence of Pb^2+^ and Ni^2+^ ions after adsorption implies that the bioadsorbents have the potential to adsorb heavy metals, while the absence of minerals such as calcium, silicon, potassium, and magnesium after adsorption have been reported previously when using orange peel [58], banana peel [53], and lemon peel [60]. The above suggests ion exchange as the adsorption mechanism.

### 3.2. Effect of the pH

**Figure 5:**
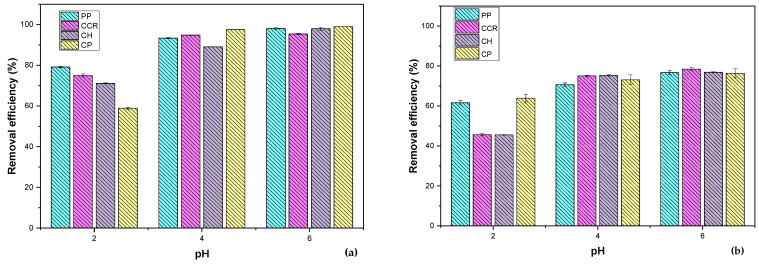
Effect of the pH of (**a**) Pb^2+^ and (**b**) Ni^2+^ using bioadsorbents from PP, CCR, CH, and CP.

The bioadsorption of heavy metals is influenced by various factors, among which pH is a critical one because the pH of the solution affects the surface charge of the biomass and the speciation of metal ions, thereby determining the electrostatic attraction and chemical interactions between the biomass and the metal ions [61]. Therefore, understanding the effect of pH on bioadsorption is essential to optimizing the process and achieving efficient removal of heavy metals. Moreover, the pH value also affects the biomass’s stability and can impact the biomass’s structural integrity, leading to variations in the biomass’s bioadsorption capacity [62]. The effect of the solution pH of Pb^2+^ and Ni^2+^ were studied in the range of 2–6 pH, as shown in Figure 5.

From the results shown in Figure 4, it is evident that the removal efficiency of the bioadsorbents is increasing while the pH augments, from 2 to 6, with the CH and CCR having the biggest enhancement of their adsorption affinity with the heavy metals. All bioadsorbents exhibited their best performance at pH 6. Thus, at a lower pH, there is a higher concentration of H^+^ in the solutions, which would compete with the Pb^2+^ and Ni^2+^ for the active centers on the surface of the adsorbents, causing a decrease in the binding between the metals and the bioadsorbent. It has been reported that the adsorption efficiency of Pb^2+^ and Ni^2+^ is enhanced when the pH is increased, and more functional groups appear for the removal of cations due to the reduction of H^+^ concentration, enhancing the adsorption of the ions [46]. Thus, the increase in removal with pH may be due to the decreasing electrostatic repulsion between the ions and the positively charged surface of the bioadsorbents at pH higher than pH_PZC_ (Figure 1); in addition, maximum elimination occurs for both ions at pH 6, where the hydrolyzed species of the ions are more soluble than the cations, considering the speciation of Pb^2+^ [63] and Ni^2+^ [64]. In the case of Pb^2+^, at pH 4–6, there is the presence of Pb^2+^, Pb(OH)_2_, and PbCl^+^, while for Ni^2+^, there is the presence of the radical as well as Ni(OH)^+^. Therefore, pH 6 was selected as the optimum value for further experiments.

### 3.3. Impact of the Particle Size, Temperature, and Adsorbent Dose

Particle size and adsorbent dose are crucial parameters affecting the bioadsorption process of heavy metals. Particle size determines the specific surface area available for adsorption [65], while adsorbent dose affects the amount of binding sites available for heavy metals [34]. A balance between these two parameters is necessary to achieve optimal bioadsorption efficiency. Understanding the effect of particle size and adsorbent dose on bioadsorption is essential for the development of effective and sustainable methods for heavy metal removal from contaminated water and wastewater. In addition, the temperature plays a crucial role in the heavy metal bioadsorption process as it affects the kinetics, thermodynamics, and stability of the adsorbent [66]. The adsorption of Pb^2+^ and Ni^2+^ in the four raw biomasses is shown in Table 4 for the experiments presented in the central composition design (Table 1). At the end of the tests, the residual amount of lead and nickel in the solution was measured, and the adsorbed quantity was calculated using Equations (1) and (2).

For the improvement of the process efficiency, the response surface methodology approach (RSM) was used because this method allows to explain the statistical main effect of the most significant parameters, its interaction with other parameters, and their quadratic effect that have impacts on the responses. Based on the application of RSM, a model was developed from the experimental data (Supplementary files: Table S1) for each system bioadsorbent-metal with the coefficient of determination (R^2^). The regression models calculated were used to explain the interactions between the coded and evaluated factors and the response.

**Table 4:** The 16 adsorption tests of the central composition design for Pb^2+^ and Ni^2+^ adsorption using PP, CCR, CH, and CP as adsorbents.

Run	Temperature, °C	Particle Size, mm	Adsorbent Dose, g	Pb^2+^ Removal (mg/g)				Ni^2+^ Removal (mg/g)			
				PP	CCR	CH	CP	PP	CCR	CH	CP
**1**	40.0	1.0	0.5	18.54	18.89	9.85	8.63	19.23	8.95	10.15	13.45
**2**	40.0	1.0	0.15	41.86	57.63	33.37	30.03	33.89	24.06	30.46	27.63
**3**	55.0	1.219	0.325	9.38	29.96	12.89	12.63	7.89	11.58	10.68	9.37
**4**	55.0	0.6775	0.03	198.01	123.78	161.03	76.91	98.05	105.37	98.45	63.89
**5**	55.0	0.6775	0.325	25.11	22.46	15.76	11.96	18.35	12.35	9.53	10.57
**6**	70.0	0.355	0.5	19.16	18.73	10.03	8.67	17.63	8.19	7.89	8.05
**7**	80.22	0.6775	0.325	23.33	18.63	13.23	14.74	19.05	11.36	10.13	12.36
**8**	70.0	0.355	0.15	47.89	56.18	33.18	25.95	26.45	21.58	18.79	21.06
**9**	40.0	0.355	0.15	29.73	39.97	33.47	30.03	26.37	24.67	21.93	19.86
**10**	70.0	1.0	0.15	46.63	47.63	33.38	58.96	23.45	21.99	26.83	29.13
**11**	40.0	0.355	0.5	14.86	19.02	10.07	9.23	11.89	7.97	8.36	7.56
**12**	29.77	0.6775	0.325	13.97	22.78	14.53	13.86	10.63	12.04	11.08	10.37
**13**	55.0	0.135	0.325	25.82	14.76	15.36	12.33	23.56	12.37	13.18	10.33
**14**	70.0	1.0	0.5	19.97	10.83	10.12	8.39	17.35	8.13	9.17	8.03
**15**	55.0	0.6775	0.619	14.13	15.35	8.15	6.97	11.36	7.98	8.04	7.06
**16**	55.0	0.6775	0.325	25.06	22.37	15.76	12.15	20.69	12.29	13.13	11.63

The model equations were tested and analyzed using the t-test and the analysis of variance (ANOVA). The ANOVA showed a 90% confidence level (Supplementary Files: Tables S2–S9), which means the regression is statistically significant for the systems CCR-Pb^2+^, CP-Ni^2+^, and CP-Pb^2+^, with the interactions AA and CC being the most influential factors when using CCR, and the temperature and the interaction CC when using CP; the above, accordingly, with a *p*-value lower than 0.05.

From the study of the temperature effect over the adsorption process, it is possible to analyze the thermodynamic behavior of the system, which involves analyzing the entropy, enthalpy, and Gibbs free energy changes during the adsorption process [67]. These thermodynamic parameters can help predict the feasibility and spontaneity of the process. Additionally, the effect of temperature on particle size and adsorbent dose must also be investigated to optimize the bioadsorption process for heavy metals. From Table 5, the negative value of the adsorption enthalpy (∆H°) indicates that the adsorption process is exothermic, as it releases energy in the bonding of the ions to the functional groups of the bioadsorbents [68]. The heat formed during the process is due to the condensation of the adsorbate plus the energy generated in the adsorbent-adsorbate bonding. The negative value of the adsorption entropy (∆S°) indicates low reversibility in the process and low randomness in the liquid-solid interface in the adsorption process [69]. Furthermore, the negative values of the Gibbs free energy (∆G°) at all evaluated temperatures indicate that the removal of the metal occurs spontaneously for the adsorption of Pb^2+^ and Ni^2+^ [70].

To investigate the temperature effect on the adsorption of Pb^2+^ and Ni^2+^ on the studied biomasses, the distribution coefficient, *K_d_* (L/g), was calculated at temperatures of 303.15, 328.15, and 353.15 K using Equation (3).

(3) $$K_{d}=\frac{q_{e}}{C_{e}}$$

The *K_d_* values obtained for the adsorption of Pb^2+^ and Ni^2+^ on the biomasses under study are presented in Table 6. The results indicate a decrease in Kd values as the temperature increased within the evaluated range (from 303.15 to 353.15 K), suggesting an exothermic adsorption of metals for all the bioadsorbents. The above is consistent with the results found for ∆H°, also shown in Table 6. Similar results were found when using Celtek clay for the removal of Pb^2+^ and Cr^3+^, showing a decrease in *K_d_* while the temperature rose from 303 to 323 K [71]. Zhang et al. [72] estimated the *K_d_* of Cu^2+^, Zn^2+^, Ni^2+^, Pb^2+^, Cd^2+^, Mn^2+^, Co^2+^, and Cr^3+^ onto poly(acrylic acid)-grafted chitosan and biochar composites to establish the adsorbent’s affinity for the adsorbate when working with multicomponent solutions. It is considered that an adsorbent with a *K_d_* value ≥ 10^4^ mL/g is exceptional [73]. They found strong selectivity for Cr^3+^ and Pb^2+^ when the amount of adsorbent was sufficient to adsorb the heavy metals in the solution. Based on our results, it could be appropriate to say that working at low temperatures would favor the adsorption process, and the bioadsorbents studied present great potential in both wastewater purification and recycling wastewater from specific industries, such as electroplating and electronic dismantling.

### 3.4. Effect of the Time and Adsorption Kinetics

The kinetics of the bioadsorption process are crucial aspects that determine the efficiency of heavy metal removal from wastewater [74]. Understanding the relationship between the time required for the adsorption process and the adsorption capacity is essential to optimizing the process. Moreover, it is necessary to study the kinetic models to determine the mechanisms involved in the adsorption process. These models provide insights into the controlling steps of the process, which can be useful in designing an efficient and cost-effective bioadsorption system [75]. The kinetic adsorption data in the present study were adjusted to pseudo-first order (PFO), pseudo-second order (PSO), Elovich, and intraparticle diffusion (ID).

**Figure 6:**
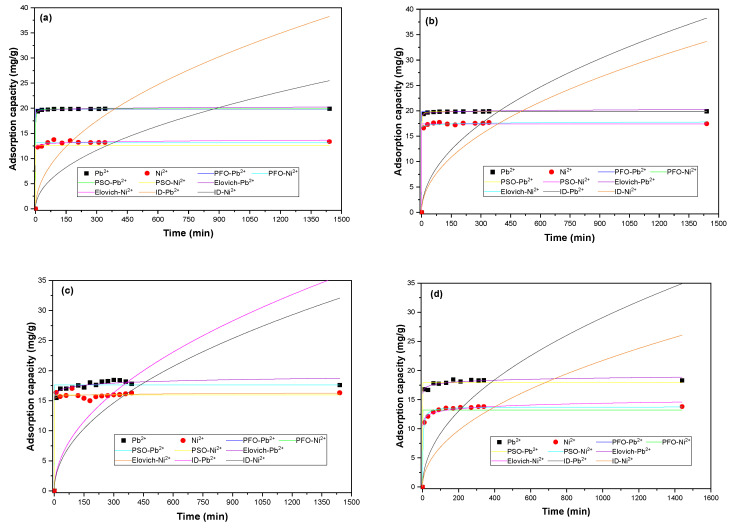
Pb^2+^ and Ni^2+^ adsorption adjustment to non-linear kinetic models using (**a**) PP, (**b**) CCR, (**c**) CH, and (**d**) CP.

From Figure 6, it is evident that the equilibrium time is reached at 120 min for Pb^2+^ and Ni^2+^ when using any of the evaluated biomasses. In addition, a rapid rate of adsorption is observed in the initial minutes of contact between the solution and the adsorbent, which is attributed to the high availability of vacant adsorption sites in the initial stages of the process [76]. From Table 6 and Table 7, it is observed that the PSO and Elovich models have the highest R^2^ and the lowest reduced Chi Square. The above refers to the proportionality between the rate of adsorption and the square of the number of unoccupied adsorption sites on the adsorbent surface, implying that the rate of adsorption decreases exponentially with increasing surface coverage [24].

A kinetics study can contribute to the improvement of heavy metal removal by providing valuable insights into the rate at which the adsorption process occurs. By understanding the kinetics, the optimal contact time between the adsorbent and the metal ions can be determined for maximum removal efficiency. This information helps in designing efficient treatment systems and optimizing operational parameters such as adsorbent dosage and flow rate, and is a useful insight into the scaling up of the adsorption process [1]. Additionally, studying the kinetics allows for the identification of the rate-limiting step in the adsorption process, which can guide further research and development efforts to enhance the removal of heavy metals. Thus, the kinetic data fitting the PSO model implies a two-step adsorption process. The first step involves metal diffusion from the solution to the external surface of the adsorbent, explaining the initial rapid adsorption rate. The second step involves the intraparticle diffusion of the metal into the pores of the bioadsorbents [2].

With the Elovich and PSO models as the best fit, it is assumed that chemisorption is the mechanism controlling the process, and due to increasing surface coverage, the rate of adsorbate elimination decreases with time. The approaching equilibrium factor (*R_E_*) determined by the Elovich model is given by Equation (4):

(4) $$R_{E}=\frac{1}{q_{e}\times \beta }$$

The similar adjustment of the PFO, PSO, and Elovich models to the experimental data could be attributed to the fact that all the models consider that the rate of adsorption depends on the initial concentration and the adsorption capacity depends on the available adsorption sites and the affinity between adsorbate and adsorbent [77]. In addition, these empirical equations assume that the concentration of the adsorbate in the solution is constant (considering a chemical reaction where one of the reactant species is in excess, with an approximately constant concentration, it has no effect on the observed rate of the reaction); they also assume that the kinetics are based on the adsorption capacity of solids instead of the concentration in the liquid phase [78]. These assumptions of the evaluated models may lead to similar adjustments when data have a similar tendency.

It is also observed from Figure 5 and Table 7 and Table 8 that the intraparticle diffusion model does not describe the data, thus having the highest reduced Chi-Square values and R^2^ equal to zero in all cases. These results can be attributed to the complexity of the adsorption process and the tendencies of the data. For further studies, we will apply this and other diffusive models to different segments of the data before and after reaching equilibrium in the system. There have been reports that diffusional models that also consider the pore volume can accurately describe the kinetic adsorption data of different pollutants present in water [79,80].

The evaluation of R_E_ parameters provides an idea about the characteristics and curvature of the associated Elovich adjustment. Thus, when R_E_ is higher than 0.3, a gradual increase in the curve is observed, indicating a limited initial adsorption rate. For values between 0.1 and 0.3, a moderate increase in the curve is reported. When the value is between 0.02 and 0.1, a rapid rise in the curve is recorded. If the value is lower than 0.02, instant equilibrium is reached [81]. All the obtained values of R_E_ were lower than 0.1, confirming the chemical nature of the system and implying strong binds between the metals and the adsorbent active centers. Regarding the q_e_ parameter of the PFO and PSO models, they are consistent with the experimental data with a low deviation. The intraparticle diffusion model did not describe the adsorption data, showing a highly Reduced Chi-Square. This could be associated with the pore diffusion mechanism as the rate-controlling step.

**Table 6:** Adjustment parameter of Pb^2+^ adsorption data to the evaluated kinetic models.

Model	Equation	Parameter	PP	CCR	CH	CP
PFO	$q_{t}=q_{e}\left(1-e^{-k_{1}t}\right)$	q_e1_ (mg/g)	19.815 ± 0.046	19.815 ± 0.046	17.607 ± 0.197	17.895 ± 0.188
		k_1_ (min^−1^)	2893.871	2892.993	7120.422	960.287
		R^2^	0.999	0.999	0.973	0.987
		Reduced Chi-Square	0.023	0.023	0.585	0.390
PSO	$q_{t}=\frac{t}{\left(\frac{1}{q_{e}^{2}k_{2}}\right)+\left(\frac{t}{q_{e}}\right)}$	$q_{e}^{2}$ (mg/g)	19.905 ± 0.009	19.906 ± 0.009	17.607 ± 0.197	17.895 ± 0.188
		k_2_ (g/mg.min)	0.187 ± 0.01	0.187 ± 0.011	1.342 × 10^21^	1.296 × 10^13^
		R^2^	0.999	0.999	0.973	0.987
		Reduced Chi-Square	6.178 × 10^−4^	6.881 × 10^−4^	0.585	0.390
Elovich	$q_{t}=\frac{1}{\beta }\ln\left(\alpha \beta \right)+\frac{1}{\beta }\ln\left(t\right)$	β (g/mg)	3.74 × 10^44^ ± 1.921	4.133 × 10^44^ ± 1.406	1.908 × 10^27^ ± 0.388	4.796 × 10^16^ ± 3.856
		α (mg/g min)	5.541 ± 0.037	5.519 ± 0.035	1.932 ± 0.388	2.472 ± 0.461
		R_E_	1.34 × 10^−46^	1.22 × 10^−46^	0.029	0.023
		R^2^	0.999	0.999	0.990	0.997
		Reduced Chi-Square	0.017	0.016	0.210	0.099
ID	$q_{t}=k_{3}t_{0.5}$	k^3^	1.008 ± 0.189	1.008 ± 0.189	0.942 ± 0.123	0.920
		R^2^	0	0	0	0
		Reduced Chi-Square	109.922	109.871	63.059	85.035

**Table 7:** Adjustment parameter of Ni^2+^ adsorption data to the evaluated kinetic models.

Model	Equation	Parameter	PP	CCR	CH	CP
PFO	$q_{t}=q_{e}\left(1-e^{-k_{1}t}\right)$	q_e1_ (mg/g)	13.112 ± 0.132	17.423 ± 0.091	15.951 ± 0.121	13.194 ± 0.262
		k_1_ (min^−1^)	521.263	1579.007	1047.909	4605.319
		R^2^	0.988	0.996	0.987	0.955
		Reduced Chi-Square	0.191	0.0091	0.219	0.755
PSO	$q_{t}=\frac{t}{\left(\frac{1}{q_{e}^{2}k_{2}}\right)+\left(\frac{t}{q_{e}}\right)}$	$q_{e}^{2}$ (mg/g)	12.573 ± 0.240	17.423 ± 0.091	15.951 ± 0.121	13.754 ± 0.084
		k_2_ (g/mg.min)	1.725 × 10^11^ ± 2.737	3.301 × 10^13^	4.737 × 10^23^	0.027 ± 0.003
		R^2^	0.967	0.997	0.987	0.997
		Reduced Chi-Square	0.519	0.091	0.219	0.049
Elovich	$q_{t}=\frac{1}{\beta }\ln\left(\alpha \beta \right)+\frac{1}{\beta }\ln\left(t\right)$	β (g/mg)	1.218 ± 2.506 × 10^24^	6.282 ± 2.235	6.003 × 10^44^ ± 4.984	2.073 × 10^37^ ± 6.497
		α (mg/g min)	4.542 ± 1.598	4.002 × 10^44^ ± 8.599	6.905 ± 0.096	1.689 ± 0.249
		R_E_	0.063	0.009	1.044 × 10^−46^	3.656 × 10^−39^
		R^2^	0.993	0.998	0.985	0.992
		Reduced Chi-Square	0.105	0.056	0.253	0.133
ID	$q_{t}=k_{3}t_{0.5}$	k_3_	0.672 ± 0.124	0.888 ± 0.166	0.845 ± 0.115	0.687 ± 0.119
		R^2^	0	0	0	0
		Reduced Chi-Square	46.824	84.692	55.615	43.518

Similar results were reported when using amidoxime manna tree residues for removing lead, having a fast adsorption rate and reaching equilibrium at 45 min of contact time [26]. Furthermore, the pomegranate resulted in a good adsorbent of Pb^2+^ present in real wastewater; the kinetic data were fitted to the Elovich model with an R^2^ value greater than 0.9 and an SSE ranging from 0 to 0.002 in all cases [24]. Ni^2+^ adsorption kinetic data were also fitted by the Elovich and PSO models when using peat, compost, brown algae, sawdust, and wood ash [30], modified zeolite [82], and aloe vera [83].

### 3.5. Effect of the Initial Concentration and Adsorption Isotherms

**Figure 7:**
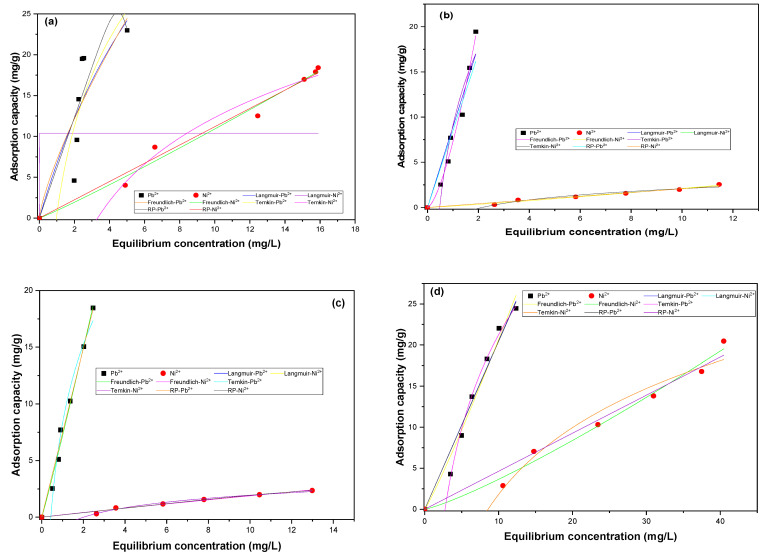
Pb^2+^ and Ni^2+^ adsorption adjustment to non-linear isotherm models using (**a**) PP, (**b**) CCR, (**c**) CH, and (**d**) CP.

The concentration of heavy metals and the corresponding adsorption isotherms are key factors that determine the effectiveness of bioadsorption techniques. The initial concentration of heavy metals in wastewater affects the capacity of the adsorbent material to remove the ions [84]. Adsorption isotherms help to determine the relationship between the amount of heavy metals adsorbed onto the adsorbent and the concentration of heavy metals remaining in the solution at equilibrium [85]. The graphical representation of the Langmuir, Freundlich, Redlich-Peterson (RP), and Temkin non-linear models can be observed in Figure 7, and the adjustment parameters in Table 8 and Table 9.

The fit to the Langmuir model indicates the formation of a monolayer during the adsorption process because the active centers are considered equal. Langmuir’s separation factor (R_L_) expresses the favorable or unfavorable nature of the process: favorable and unfavorable adsorption are assumed when R_L_ is between 0 and 1, and larger than 1, respectively. In addition, when R_L_ is equal to 0 or 1, irreversible and linear adsorption, respectively, occur [86]. The adjustment to the Freundlich model shows multilayer adsorption due to the heterogeneous surface of the adsorbent, where Freundlich’s constant (K_F_) indicates the magnitude of the equilibrium adsorption rate. The parameter n is indicative of the heterogeneity of the adsorption sites; when n is higher than 1, the adsorption would depend on the distribution of the active centers, and if n is lower than 1, the process is controlled by chemisorption [87]. Redlich-Peterson is an empirical model, a result of the combination of Langmuir and Freundlich models, and assumes that the mechanism is not described by a monolayer [88]. The Temkin model assumes the linear diminution of the affinity between adsorbate and adsorbent as time passes, and it is valid for an intermediate range of initial concentration [89].

From Figure 7, it is established that the adsorption capacity of the bioadsorbents increases with the initial concentration, which is related to the growth in the concentration-induced driving force [90]. Considering the R^2^ and Reduced Chi-Square values reported in Table 8, it can be said that the Pb^2+^ equilibrium on PP is not well described by any evaluated model. Thus, it could be due to the complex interaction between the metal and the active centers of the biomass. This lack of adjustment could lead to the development of a generalized model based on the phenomenology of the adsorption process in a solid-liquid system. Temkin exhibited the best fit when using CCR as an adsorbent, which indicates that the controlling mechanism of the process is chemisorption by the binding between the Pb^2+^ and the available adsorption sites [90]. The equilibrium data of CH and CP are well described by the Freundlich model, assuming that the process is non-ideal because of the energy difference between the adsorbent sites and reversible with the formation of a multilayer. The parameter n is higher than 1 for PP and CCR, indicating that the adsorption of Pb^2+^ onto this biomass depends on the distribution of the active centers. When using CH and CP, the n is lower than 1, showing that the process is controlled by chemisorption [91]. From the value obtained for R_L_, it can be said that the adsorption of Pb^2+^ onto the four dry evaluated biomasses is favorable.

**Table 8:** Fitting parameters of Pb^2+^ adsorption on the bioadsorbent to non-linear isotherm models.

Model	Equation	Parameter	PP	CCR	CH	CP
Freundlich	$q_{e}=K_{F}C_{e}{}^{1/n}$	K_F_	7.167 ± 2.858	10.244 ± 2.453	7.185 ± 0.408	1.646 ± 0.459
		n	1.312 ± 0.574	1.949 ± 0.397	0.947 ± 0.069	0.910 ± 0.103
		R^2^	0.718	0.892	0.988	0.997
		Reduced Chi-Square	24.827	21.825	0.619	0.023
Langmuir	$q_{e}=\frac{\left(q_{\max}K_{L}C_{e}\right)}{1+K_{L}C_{e}}$	q_max_	699.267 ± 0.8903	568.794 ± 8.769	101.535 ± 7.212	116.820 ± 1.028
		K_L_	0.106 ± 0.179	0.159 ± 0.055	7.341 × 10^–4^ ± 0.052	1.76 × 10^−4^ ± 0.001
		R_L_	0.086	0.059	0.932	0.983
		R^2^	0.726	0.936	0.987	0.973
		Reduced Chi-Square	24.044	13.005	0.691	2.727
RP	$q_{e}=\frac{\left({\mathrm{AC}}_{e}\right)}{1+{\mathrm{BC}}_{e}{}^{g}}$	A	6.159 ± 1.775	6.759 ± 2.172	7.465 ± 0	2.335 ± 0
		B	3.042 × 10^−9^ ± 2.499 × 10^−5^	0.019 ± 0.067	0.003 ± 0	0.143 ± 0
		g	11.508	1.631 ± 1.124	29.991 ± 0.005	1.131 ± 0
		R^2^	0.756	0.949	0.987	0.973
		Reduced Chi-Square	26.840	13.655	0.862	3.411
Temkin	$q_{e}=\frac{RT}{b_{T}}\ln\left(A_{T}C_{e}\right)$	*A_T_*	1.031 ± 0.419	1.261 ± 0.287	2.288 ± 0.193	0.363 ± 0.011
		*b_T_*	162.741 ± 64.825	183.041 ± 20.771	251.474 ± 18.875	152.928 ± 4.495
		B	15.487	13.769	10.022	16.481
		R^2^	0.757	0.951	0.981	0.967
		Reduced Chi-Square	21.388	9.864	0.986	4.256

Regarding Ni^2+^ adsorption, the Freundlich model describes the adsorption on CH, Langmuir on CCR and CP, and RP on PP. According to Freundlich’s n parameter, the adsorption on CCR depends on the distribution of the active centers, while on PP, CP, and CH, the mechanism is controlled by chemisorption. From the value obtained for R_L_, it can be said that the adsorption of Ni^2+^ onto the CCR, CH, and CP is favorable.

**Table 9:** Fitting parameters of Ni^2+^ adsorption on the bioadsorbent to non-linear isotherm models.

Model	Equation	Parameter	PP	CCR	CH	CP
Freundlich	$q_{e}=K_{F}C_{e}{}^{1/n}$	K_F_	0.919 ± 0.322	0.153 ± 0.036	0.215 ± 0.044	0.230 ± 0.091
		n	0.931 ± 0.115	0.876 ± 0.081	1.061 ± 0.101	0.833 ± 0.079
		R^2^	0.981	0.985	0.998	0.986
		Reduced Chi-Square	1.189	0.015	0.015	0.920
Langmuir	$q_{e}=\frac{\left(Q_{\max}K_{L}C_{e}\right)}{1+K_{L}C_{e}}$	q_max_	10.402 ± 2.65	26.984 ± 5.529	18.883 ± 9.598	21.615 ± 5.021
		*K_L_*	6.648 × 10^17^	7.717 × 10^−5^ ± 0.0016	0.011 ± 0.013	2.146 × 10^−5^ ± 0.005
		R_L_	1.504 × 10^−20^	0.992	0.047	0.998
		R^2^	0.329	0.979	0.082	0.976
		Reduced Chi-Square	42.348	0.021	0.014	1.602
RP	$q_{e}=\frac{\left({\mathrm{AC}}_{e}\right)}{1+{\mathrm{BC}}_{e}{}^{g}}$	A	1.194	0.456 ± 0	0.199 ± 0.019	0.641 ± 0
		B	0.068	1.204 ± 0	1.502 × 10^−6^ ± 4.675 × 10^−5^	0.382 ± 0
		g	2.256	0.234	1.644 ± 0.293	0.523 ± 0
		R^2^	0.979	0.979	0.986	0.976
		Reduced Chi-Square	1.586	0.026	0.016	1.997
Temkin	$q_{e}=\frac{\mathrm{RT}}{b_{T}}\ln\left(A_{T}C_{e}\right)$	A_T_	0.301 ± 0.036	0.466 ± 0.0.57	0.497 ± 0.037	0.117 ± 0.013
		b_T_	225.792 ± 21.669	1864.319 ± 20.771	2801.54 ± 120.649	214.655 ± 21.384
		B	11.192	1.352	0.899	11.742
		R^2^	0.977	0.965	0.989	0.970
		Reduced Chi-Square	1.470	0.034	0.0094	1.951

Table 10 shows the Langmuir parameter q_max_ data reported for removing Pb^2+^ and Ni^2+^ with adsorbents of different natures, finding that the results obtained in the present study are the average for bioadsorbents of lignocellulosic origin when removing Ni^2+^, while in the case of Pb^2+^, the results excel the other reported adsorbents in the literature.

### 3.6. Adsorption Mechanism

The adsorption mechanism plays a crucial role in the efficiency of heavy metal removal by bioadsorbents. Understanding the mechanism could help optimize the process and enhance the adsorption capacity. The mechanism is influenced by the intrinsic factors of the adsorbents as well as the properties of the pollutant [93]. In addition, studying the adsorption equilibrium and kinetics can help evaluate the adsorption capacity and predict the performance of the bioadsorbent under different conditions.

Through the characterization shown in Chapter 3.1, the kinetic study in Chapter 3.5, and the isotherm adjustment in Chapter 3.6, it can be said that the adsorption of Pb^2+^ and Ni^2+^ onto the dry biomasses evaluated as bioadsorbents can be controlled by the following mechanisms: chelation, intra-molecular dispersion, dispersion of the ions from the bulk to the adsorbent surface, and intraparticle diffusion. Those mechanisms involve the existence of a driving force that allows the movement of the ions from the solution, the dispersion through the boundary to the surface of the adsorbent, the fixation in the available sites at the surface, and then the movement to the insight pores. Similar mechanisms were reported when using oximated manna in the removal of Pb^2+^ in batch systems [26]. For the removal of Ni^2+^ onto biochars from crops, weeds, and trees, the mechanism reported was chelation by the occurrence of redox reactions and the formation of bonds with H on the surface of the bioadsorbents [94].

### 3.7. Bioadsorption: Future Path

Bioadsorption offers several advantages over conventional methods, including cost-effectiveness, easy availability, sludge-free operation, regenerability, and technological feasibility. It exhibits a strong affinity for heavy metal removal and enables the recovery of metals from the adsorbent matrix. The study of isotherms and kinetics reveals that the sorption rate depends on the physical and chemical properties of the sorbent as well as parameters like pH, time, adsorbent dosage, and temperature. The removal rate of metal ions typically increases over time until equilibrium is reached between the solid and liquid phases. Recent literature suggests that adsorption technology is a superior alternative to traditional methods for extracting toxic metals. Furthermore, including more active functional groups on the adsorbent surface is essential to effectively remove a wide range of contaminants and maintain long-term mobility, meeting the requirements for practical wastewater treatment. To enhance toxin removal efficiency against diverse contaminants, dynamic compounds with high affinity have been utilized to modify the adsorbent surface.

The study of the kinetics and equilibrium of adsorption is important for simulating the adsorption process in specialized software such as Aspen Adsorption and Unisim. In addition, understanding the mechanism of adsorption through experimental data has an important role in scaling the process for industrial purposes. For both goals, optimization through tools like response surface methodology is an important step in order to carry out the experiments in the best conditions for maximizing the removal of the targeted pollutant. The above, considering that the adjustment to the models can be applied for adsorption/desorption sequences in packed bed systems. Further, the models can describe the non-linear behavior of industrial systems.

The mathematical modeling of the adsorption process in a liquid-solid system from a phenomenological point of view can contribute to understanding the interactions, especially at the boundary, and how the different factors interact. The developed models can be proposed for industrial approaches and for analyzing different operating conditions for optimization purposes [95].

## 4. Conclusions

This study reported the successful evaluation of raw biomasses of plantain peels (PP), corn cob residues (CCR), cocoa husk (CH), and cassava peels (CP) as adsorbents of Pb^2+^ and Ni^2+^ in aqueous solution. Results from the FTIR and SEM-EDX analyses confirm the presence of functional groups and the presence of the removed heavy metals on the adsorbent surface after the process. It was evidenced that electrostatic attraction, cation exchange, and surface complexation were the main mechanisms involved in the adsorption of metals onto the bioadsorbents. adsorbent dosage of 0.03 g, 30 °C, and a particle size of 0.325 mm. The removal efficiency of Pb^2+^ and Ni^2+^ increased with higher adsorbent dosage, with Pb^2+^ exhibiting greater biosorption capacity than Ni^2+^. The experimental isotherm of Pb^2+^ when using CH and CP is well described by the Freundlich model and Temkin for CCR. In the case of Ni^2+^, Freundlich describes the adsorption on CH, Langmuir on CCR and CP, and RP on PP. The kinetics were adjusted by PSO and Elovich models for both ions, with the adsorption rate following the order: CH > CP > CCR > PP for Pb^2+^ and CH > CCR > PP > CP for Ni^2+^. Similarly, the thermodynamic parameters are typical of an exothermic process, with low reversibility and spontaneity. It can be said that the adsorption mechanism is controlled by chemisorption and strong binding between the adsorbent and heavy metal. These findings suggest that the dry raw biomasses of plantain peels, corn cob residues, cocoa husk, and cassava peels can effectively remove lead and nickel, but further research is needed to explore their application in industrial-scale and continuous systems, which can be done by using computational tools like artificial neural networks, machine learning, and software such as Aspen Adsorption, HYSYS, or UniSim.

## Figures and Tables

**Table 1:** Central composition design for Pb^2+^ and Ni^2+^ adsorption on the bioadsorbents under study.

Independant Factors	Symbol	Unit	Range and Level				
			−α	−1	0	+1	+α
Particle size	A	mm	0.135	0.355	0.677	1	1.22
Adsorbent quantity	B	g	0.0031	0.15	0.325	0.5	0.619
Temperature	C	°C	30	40	55	70	80

**Table 2:** Acid groups on the surface of the bioadsorbents.

Bioadsorbent	Acid Groups (mmol/g)			Total Acid (mmol/g)	Total Basic (mmol/g)
	Carboxylic	Lactones	Phenolic		
PP	2.362	0	1.889	4.251	0.555
CCR	4.235	0	1.889	6.124	0.692
CH	3.798	0	1.687	5.485	0.692
CP	4.185	0	1.828	6.013	0.696

**Table 3:** EDS results of weight percentage quantification of the bioadsorbents under study before and after Pb^2+^ and Ni^2+^ adsorption.

Biomass/Element	C	O	Si	Ca	Cu	P	Al	K	S	Fe	Mg	Pb	Ni
PP	50.90	44.60	3.87	0.19	0.45								
PP-Pb^2+^	61.12	37.18	0.48		0.31							0.91	
PP-Ni^2+^	56.87	41.44	0.66		0.22								0.81
CCR	48.08	37.81	8.19			5.53		0.39					
CCR-Pb^2+^	38.17	56.37	0.87			0.47						3.39	
CCR-Ni^2+^	55.84	42.64	0.26			0.13		0.42					0.7
CH	47.57	45.10		0.20	0.26	0.11	0.35	5.84	0.32		0.26		
CH-Pb^2+^	57.78	38.94				0.16	0.38	1.42				1.32	
CH-Ni^2+^	16.99	67.23		5.04			1.31	4.01	0.47		2.24		0.83
CP	66.27	29.97	1.08	0.63			0.46	0.83	0.32	0.43			
CP-Pb^2+^	51.20	41.45	0.77	0.41			0.45	0.34		0.44		0.99	
CP-Ni^2+^	55.55	36.18	3.03	0.56			1.52	0.54	0.21	1.19		1.22	

**Table 5:** Thermodynamic parameters for the adsorption of Pb^2+^ and Ni^2+^.

Biomass	T (K)	Pb^2+^				Ni^2+^			
		K_d_	ΔG° (KJ/mol)	ΔH° (KJ/mol*K)	ΔS° (KJ/mol)	K_d_	ΔG° (KJ/mol)	ΔH° (KJ/mol*K)	ΔS° (KJ/mol)
PP	303.15	2695	−1.439	−0.057	−0.047	3023	−5.0135	−4.335	−0.023
	328.15	1896	−2.611	−	−	2016	−5.588		
	353.15	938	−3.783	−	−	1350	−6.163		
CCR	303.15	2911	−3.891	−4.065	−0.026	3230	−8.799	−7.499	−0.053
	328.15	1266	−4.443			2880	−9.931		
	353.15	966	−4.993			1980	−11.057		
CH	303.15	2450	−0.549	−2.121	−0.143	3429	−9.199	−11.290	−0.070
	328.15	1567	−0.782			2828	−7.428		
	353.15	1010	−12.837			1832	−5.657		
CP	303.15	1549	−6.337	−9.737	−0.077	4050	−7.345	−0.208	−0.058
	328.15	1203	−8.405			3820	−9.794		
	353.15	983	−12.476			2596	−13.466		

**Table 10:** Comparison of Langmuir’s parameter q_max_ for adsorbents of different natures.

Heavy Metals	Adsorbent	q_max_ (mg/g)	Reference
Pb^2+^	Wood derived biochar	0.44	[24]
	Groundnut modified with guar gum	0.55	[46]
	*Phragmites*	5.46	[65]
	Rice husk nanocellulose	6.1	[84]
	Commercial activated carbon	16.84	[74]
	Phosphoric acid-modified biochar from chicken feather	24.41	[59]
	*Callinectes sapidus* biomass	31.44	[92]
	Nitrile amidoximation of manna tree	73.25	[26]
	Breadfruit peel	85.42	[85]
	Date palm biochar	146	[27]
	Cocoa husk	101.54	Present study
	Cassava peels	116.82	
	Corncob residues	568.79	
	Plantain peels	699.27	
Ni^2+^	Groundnut modified with guar gum	0.74	[46]
	Compost	8.8	[30]
	Aloe vera leaves powder	10	[83]
	Plantain peels	10.40	Present study
	Cocoa husk	18.88	
	Cassava peels	21.62	
	Corncob residues	26.98	
	Carbon from cassava peels activated with KOH	41.41	[29]
	Carbon from cassava peels activated with H_3_PO_4_	47.39	
	Carbon from cassava peels	35.34	
	Cassava peels	34.48	
	Zeolite modified with magnesium hydroxide	25.41	[82]
	Bentonite	23.93	[69]
	Peat moss	22	[30]

## Data Availability

The data presented in this study are available upon reasonable request from the corresponding author.

## Supplementary Materials

The following supporting information can be downloaded at: https://www.mdpi.com/article/10.3390/ma16134884/s1, Figure S1. Pictures of dry biomasses used as precursors: (a) PP, (b) CCR, (c) CH, and (d) CP; Table S1. Statistical analysis of experimental data for determining optimal conditions; Table S2. Model fitting and regression analysis of Pb^2+^ adsorption process onto PP; Table S3. Model fitting and regression analysis of Ni^2+^ adsorption process onto PP; Table S4. Model fitting and regression analysis of Pb^2+^ adsorption process onto CCR; Table S5. Model fitting and regression analysis of Ni^2+^ adsorption process onto CCR; Table S6. Model fitting and regression analysis of Pb^2+^ adsorption process onto CH; Table S7. Model fitting and regression analysis of Ni^2+^ adsorption process onto CH; Table S8. Model fitting and regression analysis of Pb^2+^ adsorption process onto CP; Table S9. Model fitting and regression analysis of Ni^2+^ adsorption process onto CP.
