# Supplementary material for: High-Efficiency Removal of Lead and Nickel Using Four Inert Dry Biomasses: Insights into the Adsorption Mechanisms
Source: Materials (Basel). 2023 Jul 7;16(13):4884. doi: 10.3390/ma16134884 (PMC10343542; doi:10.3390/ma16134884)
Supplement: Supplementary file 1 [file materials-16-04884-s001.zip › materials-2452937-supplementary.pdf]

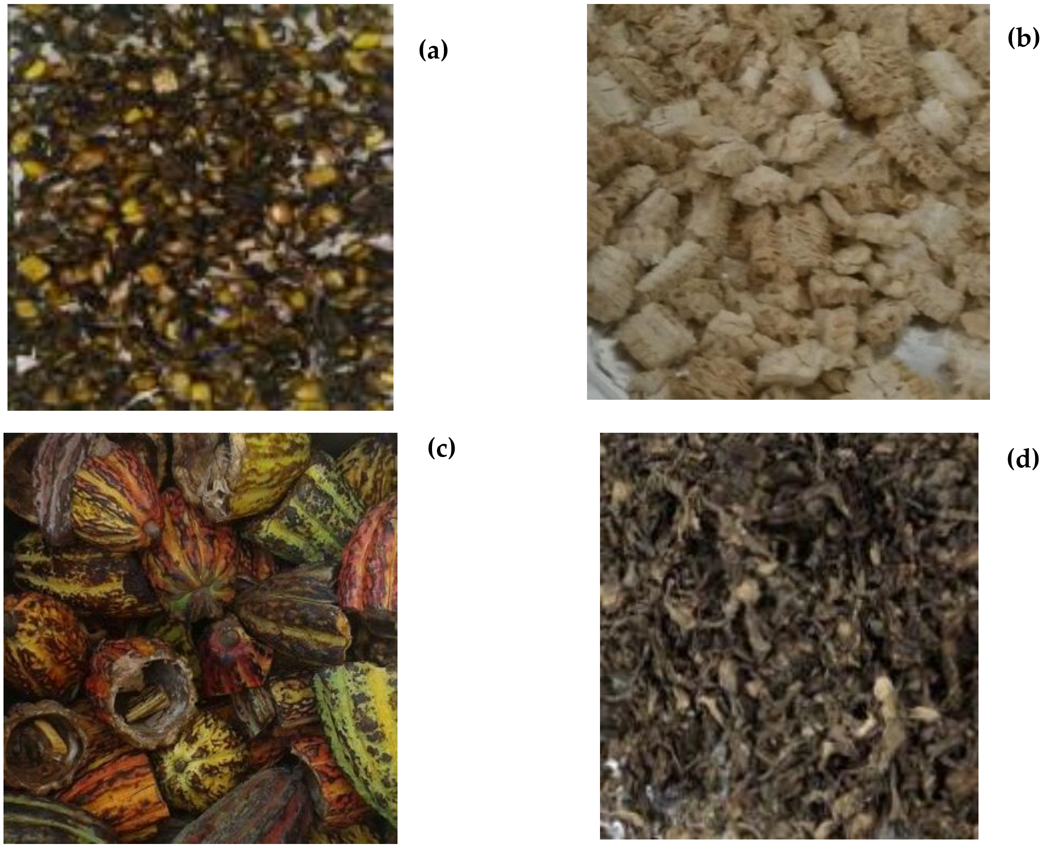

Figure S1. Pictures of dry biomasses used as precursors: (a) PP, (b) CCR, (c) CH, and (d) CP.

Table S1. Statistical analysis of experimental data for determining optimal conditions.

Biomass	Equation	R^2 (%)
PP-Pb^{2+}	$Y = 1.4951 + 4.01281A + 116.329B - 628.639C + 0.0295033A^2 - 0.42155AB - 0.819048AC - 67.4917B^2 - 14.1307BC + 792.337C^2$	75.43
PP-Ni^{2+}	$Y = 22.8737 + 1.72026A + 58.7792B - 350.646C - 0.0144131A^2 - 0.468734AB + 0.677143AC - 28.1782B^2 + 5.62569BC + 354.298C^2$	74.40
CCR-Pb^{2+}	$Y = 36.2144 + 1.61341A + 81.4304B - 398.046C - 0.00761609A^2 - 0.878036AB - 0.693333AC - 10.8917B^2 - 37.9623BC + 507.353C^2$	91.88
CCR-Ni^{2+}	$Y = 41.7371 + 1.35349A + 36.7221B - 380.846C - 0.0132499A^2 - 0.000516796AB + 0.217143AC - 27.7891B^2 + 2.48062BC + 421.758C^2$	76.36
CH-Pb^{2+}	$Y = 55.0954 + 2.23043A + 57.5469B - 579.714C - 0.0205458A^2 + 0.0157623AB + 0.0242857AC - 43.707B^2 - 0.509413BC + 665.159C^2$	77.21
CH-Ni^{2+}	$Y = 40.9395 + 1.07185A + 40.1246B - 351.665C - 0.0107697A^2 - 0.0258398AB + 0.253333AC - 18.8118B^2 - 29.9003BC + 412.934C^2$	79.54
CP-Pb^{2+}	$Y = 67.5747 - 0.334576A - 14.4801B - 195.855C + 0.00247902A^2 + 0.86124AB - 1.22143AC - 0.797223B^2 - 75.0609BC + 336.586C^2$	93.71
CP-Ni^{2+}	$Y = 29.1382 + 0.456383A + 32.9299B - 200.809C - 0.00213319A^2 - 0.144961AB + 0.363333AC - 9.75633B^2 - 22.0819BC + 262.345C^2$	88.90

Table S2. Model fitting and regression analysis of Pb^{2+} adsorption process onto PP.

Source	Sum of squares	DF	Mean Square	F-Value	P-Value
A: Particle size	31.3036	1	31.3036	0.03	0.8785
B: Adsorbent quantity	4.23605	1	4.23605	0.00	0.9551
C: Temperature	5451.83	1	5451.83	4.43	0.0799
AA	408.154	1	408.154	0.33	0.5855
AB	33.0485	1	33.0485	0.03	0.8752
AC	36.98	1	36.98	0.03	0.8680
BB	455.411	1	455.411	0.37	0.5652
BC	5.08805	1	5.08805	0.00	0.9508
CC	5467.1	1	5467.1	4.44	0.0796
Total error	7380.24	6	1230.04		
Total (corr.)	30041.8	15			

Table S3. Model fitting and regression analysis of Ni^{2+} adsorption process onto PP.

Source	Sum of squares	DF	Mean Square	F-Value	P-Value
A: Temperature	33.4775	1	33.4775	0.03	0.8785
B: Particle size	116.04	1	116.04	0.09	0.7766
C: Adsorbent dose	375.525	1	375.525	0.29	0.6125
AA	217.059	1	217.059	0.16	0.6988
AB	2333.98	1	2333.98	1.77	0.2314
AC	829.864	1	829.864	0.63	0.4575
BB	2146.49	1	2146.49	1.63	0.2488
BC	1.94085	1	1.94085	0.00	0.9706
CC	108.599	1	108.599	0.08	0.7836
Total error	7898.78	6	1316.46		
**Total (corr.)**	1.3842.2	15			

Table S4. Model fitting and regression analysis of Pb^{2+} adsorption process onto CCR.

Source	Sum of squares	DF	Mean Square	F-Value	P-Value
A: Particle size	24.9819	1	24.9819	0.15	0.7075
B: Adsorbent quantity	34.575	1	34.575	0.21	0.6597
C: Temperature	2234.93	1	2234.93	13.86	0.0098
AA	27.1986	1	27.1986	0.17	0.6956
AB	144.33	1	144.33	0.89	0.3807
AC	26.4992	1	26.4992	0.16	0.6993
BB	11.8603	1	11.8603	0.07	0.7953
BC	36.7224	1	36.7224	0.23	0.6501
CC	2241.6	1	2241.6	13.90	0.0098
Total error	967.663	6	161.277		
Total (corr.)	11918.2	15			

Table S5. Model fitting and regression analysis of Ni^{2+} adsorption process onto CCR.

Source	Sum of squares	DF	Mean Square	F-Value	P-Value
A: Particle size	3.24969	1	3.24969	0.01	0.9243
B: Adsorbent quantity	0.204999	1	0.204999	0.00	0.9809
C: Temperature	1541.87	1	1541.87	4.66	0.0742
AA	82.321	1	82.321	0.25	0.6356
AB	0.00005	1	0.00005	0.00	0.9997
AC	2.5992	1	2.5992	0.01	0.9322
BB	77.2058	1	77.2058	0.23	0.6461
BC	0.1568	1	0.1568	0.00	0.9833
CC	1549.04	1	1549.04	4.68	0.0737
Total error	1984.63	6	330.771		
Total (corr.)	8404.36	15			

Table S6. Model fitting and regression analysis of Pb^{2+} adsorption process onto CH.

Source	Sum of squares	DF	Mean Square	F-Value	P-Value
A: Particle size	0.231425	1	0.231425	0.00	0.9868
B: Adsorbent quantity	0.0000108875	1	0.0000108875	0.00	0.9999
C: Temperature	3837.09	1	3837.09	4.91	0.0685
AA	197.938	1	197.938	0.25	0.6326
AB	0.0465125	1	0.0465125	0.00	0.9941
AC	0.0325125	1	0.0325125	0.00	0.9951
BB	190.987	1	190.987	0.24	0.6385
BC	0.0066125	1	0.0066125	0.00	0.9978
CC	3852.91	1	3852.91	4.93	0.0681
Total error	4686.42	6	781.07		
Total (corr.)	20566.5	15			

Table S7. Model fitting and regression analysis of Ni^{2+} adsorption process onto CH.

Source	Sum of squares	DF	Mean Square	F-Value	P-Value
A: Particle size	3.24969	1	3.24969	0.01	0.9243
B: Adsorbent quantity	0.204999	1	0.204999	0.00	0.9809
C: Temperature	1541.87	1	1541.87	4.66	0.0742
AA	82.321	1	82.321	0.25	0.6356
AB	0.00005	1	0.00005	0.00	0.9997
AC	2.5992	1	2.5992	0.01	0.9322
BB	77.2058	1	77.2058	0.23	0.6461
BC	0.1568	1	0.1568	0.00	0.9833
CC	1549.04	1	1549.04	4.68	0.0737
Total error	1984.63	6	330.771		
Total (corr.)	8404.36	15			

Table S8. Model fitting and regression analysis of Pb^{2+} adsorption process onto CP.

Source	Sum of squares	DF	Mean Square	F-Value	P-Value
A: Particle size	83.3079	1	83.3079	1.34	0.2916
B: Adsorbent quantity	146.268	1	146.268	2.35	0.1764
C: Temperature	986.4	1	986.4	15.82	0.0073
AA	2.88165	1	2.88165	0.05	0.8369
AB	138.861	1	138.861	2.23	0.1862
AC	82.2403	1	82.2403	1.32	0.2944
BB	0.0635422	1	0.0635422	0.00	0.9756
BC	143.567	1	143.567	2.30	0.1799
CC	986.576	1	986.576	15.83	0.0073
Total error	373.999	6	62.3332		
Total (corr.)	5947.49	15			

Table S9. Model fitting and regression analysis of Ni^{2+} adsorption process onto CP.

Source	Sum of squares	DF	Mean Square	F-Value	P-Value
A: Particle size	7.06402	1	7.06402	0.12	0.7370
B: Adsorbent quantity	12.0286	1	12.0286	0.21	0.6623
C: Temperature	597.749	1	597.749	10.48	0.0178
AA	2.13374	1	2.13374	0.04	0.8530
AB	3.93401	1	3.93401	0.07	0.8017
AC	7.27711	1	7.27711	0.13	0.7332
BB	9.51646	1	9.51646	0.17	0.6971
BC	12.4251	1	12.4251	0.22	0.6572
CC	599.356	1	599.356	10.51	0.0177
Total error	342.322	6	57.0536		
Total (corr.)	3082.76	15			
